# Preoperative PET or PET/CT for malignant diagnosis of Gastrointestinal stromal tumors: a systematic review and meta-analysis

**DOI:** 10.1186/s12885-025-15278-3

**Published:** 2026-03-31

**Authors:** Hao-lin Li, Hua-de Huo, Shu Wang, Jian-jun Yang

**Affiliations:** 1 Department of Digestive Surgery, Xijing Hospital of Digestive Diseases, Fourth Military Medical University, Xi’an, 710032 China; 2 State Key Laboratory of Holistic Integrative Management of Gastrointestinal Cancers and National Clinical Research Center for Digestive Diseases, Xijing Hospital of Digestive Diseases, Fourth Military Medical University, Xi’an, 710032 China

**Keywords:** Gastrointestinal stromal tumors, Positron-emission tomography, Positron emission tomography-computed tomography, Diagnosis, Meta-analysis

## Abstract

**Background:**

Gastrointestinal stromal tumors (GISTs) are the most prevalent mesenchymal tumors of the gastrointestinal system; however, preoperative imaging for the accurate diagnosis of malignancy is unavailable. We conducted a meta-analysis to comprehensively confirm the value of preoperative Positron emission tomography **(**PET) in diagnosing the malignant potential of GISTs, aiming to guide preoperative preparation when biopsy is confined and prognostic evaluation for patients with localized primary GISTs after curative resection.

**Methods:**

PubMed, Web of Science, Cochrane Library, and Embase databases were systematically searched from database inception till June 1, 2025, to retrieve articles reporting the predictive value of preoperative PET or PET/CT for GISTs. Overall sensitivity (SEN) and specificity (SPE) with 95% confidence intervals (CI) using forest plots were estimated. The chi-square and I^2^ tests were used to analyze significant heterogeneity. QUADAS-2 and Deek funnel plots were constructed to assess publication bias risk. Subgroup analyses were performed to investigate the sources of heterogeneity.

**Results:**

Fifteen studies including 568 patients with GISTs were included. The findings demonstrated overall SEN (0.86, 95% CI: 0.74–0.93), SPE (0.87, 95% CI: 0.75–0.94), positive likelihood ratio 6.80, 95% CI: 3.09–14.96), negative likelihood ratio (0.16, 95% CI: 0.07–0.34), and the area under the receiver operating characteristics curve (0.93, 95% CI: 0.91–0.95). Subgroup analysis indicated that with cut-off values at ≥ 5, the diagnosis could have a higher accuracy (sensitivity, specificity, and area under the curve values were 0.89, 0.92, and 0.96, respectively).

**Conclusion:**

Preoperative PET or PET/CT exhibited excellent diagnostic performance in predicting malignancy in GISTs and may be employed as an effective non-invasive imaging device for guiding preoperative preparation when biopsy is not possible and prognostic analysis for patients with localized primary GISTs who underwent radical surgery.

**Trial registration:**

Our protocol was registered in PROSPERO (CRD42024545100) on May 9th, 2024.

**Supplementary Information:**

The online version contains supplementary material available at 10.1186/s12885-025-15278-3.

## Background

Gastrointestinal stromal tumors (GISTs), originating from the interstitial cells of Cajal (ICC), are the most common mesenchymal tumor of the gastrointestinal tract, with the stomach and small intestine (60–65% and 20–25%), respectively/being the most common. The annual incidence of GISTs is approximately 1.2 per 10^5^ individuals [1]. The malignant potential of GISTs ranges from benign tumors to rapidly progressing neoplasms [2]. Furthermore, its risk classification criteria, revised by the US National Institutes of Health (NIH) include tumor size, mitotic count, and primary site [3]. The European Society for Medical Oncology (ESMO) [4] and the National Comprehensive Cancer Network (NCCN) [5] recommended computed tomography (CT), magnetic resonance imaging (MRI), and Positron emission tomography **(**PET) for preoperative examinations. Because GISTs typically exhibit extraluminal growth, with characteristics varying on CT according to size and primary location. These tumors present as soft tissue masses with either a circular or irregular morphology, and larger masses may display heterogeneous density attributable to necrosis, cystic degeneration, or hemorrhage. Due to its high anatomical resolution, CT is of great value in the diagnosis of GISTs. However, it also has limitations, such as the potential for missed diagnoses in cases of small lesions. PET reveals varying degrees of tracer uptake in GISTs, several kinds of radiotracers have been confirmed to have great tumor affinity and low side effects with GISTs, including 18F-fluorodeoxyglucose (^18^F-FDG), 18F-fibroblast activation protein inhibitor-42 (^18^F-FAPI-42), 68Ga-fibroblast activation protein inhibitor-04 (68Ga-FAPI-04), 68Ga-1,4,7-triazacyclononane-N, N9,N99-triaceticacid-D-Phe-Gln-Trp-Ala-Val-Gly-His-Sta-Leu-NH2(^68^Ga-RM26) and so on [6, 7]. The abnormal metabolism of ^18^F-FDG facilitates the detection of small lesions that might be overlooked by CT. Besides, due to a portion of GISTs lesions show negative FDG uptake, other tracers shown greater sensitivity in predicting malignant potential and prognostic value [7]. Furthermore, no imaging modality can provide a detailed diagnosis of mitotic count and risk classification before surgery. Although the mitotic index can be obtained before surgery using endoscopic ultrasonography (EUS) or biopsy, some tumors are difficult to biopsy, the quantity of biopsied tissue is minimal for mitotic counting, and tumor metastasis secondary to hemorrhage may occur [8]. Therefore, the preoperative assessment of the malignant potential of GISTs is difficult.

GISTs were often ignored in the early stage due to the lack of characteristic clinical manifestations, so metastasis is usually present at the time of diagnosis. Burkill et al. [9] reported that the metastasis rate of malignant GISTs reached 61% at the time of diagnosis. ^18^F-FDG PET/CT is a noninvasive and convenient imaging technique, that indicates a tumor with high metabolism and malignant potential through increased FDG accumulation [10]. Preoperative PET whole-body imaging can effectively display the primary lesion and possible metastatic lesion, and help to judge the scope of the lesion, which recognize for the high-risk groups and determine the indications for treatment and the appropriate surgical procedure for resection. Additionally, ^18^F-FDG PET can help in evaluating tumor glycolysis associated with mitotic counts in multiple tumors, including GISTs [11]. Therefore, preoperative PET findings may contribute to predicting recurrence-free survival (RFS) compared with the current guidelines. In addition, because tumor glycolysis can be easily evaluated using volumetric parameters like metabolic tumor volume (MTV) or total lesion glycolysis (TLG), whereas mitotic counts are heterogeneous in larger GISTs [12, 13], preoperative PET may offer better prognostic value in predicting RFS. Recent studies indicate that PET/CT is valuable for differentiating between malignant and benign GISTs, indicating its significant role in predicting malignant potential.

However, most available data were derived from single-center studies or retrospective studies with small sample sizes. Therefore, we performed a diagnostic meta-analysis to determine the potential diagnostic value of PET in predicting the malignant potential of GISTs and to provide clinical and practical guidance.

## Methods

### Literature search

Our protocol was registered in PROSPERO (CRD42024545100) at https://www.crd.york.ac.uk/prospero/. This meta-analysis followed the Preferred Reporting Items for Systematic Evaluation (PRISMA) and Meta-Analysis statement for preferred reporting items (Additional file 1).

### Search strategy

To identify relevant publications, systematic searches were done in PubMed, Web of Science, the Cochrane Library, and Embase from database inception to June 1, 2025. The search strategy involved using Mesh Terms and related entry terms: “Gastrointestinal Stromal Tumors” AND “Positron-Emission Tomography” OR “Positron Emission Tomography Computed Tomography”. The reference lists of all included studies were analyzed to prevent the loss of relevant articles. The search strategy was adjusted to the retrieval formats of each database.

### Study selection

Subjects were recruited with the following inclusion criteria: (1) all patients in the case group were diagnosed according to clinically recognized criteria(using NIH criteria or modified NIH criteria), with the high-risk group considered positive and the other groups considered negative; (2) the intervention involved diagnosing the malignant potential of GISTs using PET or PET/CT; (3) false positive (FP), true positive (TP), false negative (FN), and true negative (TN) could be directly derived or calculated from the literature.studies; and (4) involved patients with localized primary GISTs undergoing initial preoperative evaluation. The exclusion criteria include (1) non-human trials; (2) non-case-control studies; (3) reviews, case reports, or conference abstracts; (4) insufficient data; (5) studies without postoperative histopathology; and (6) studies focused solely on recurrence, restaging, or treatment response monitoring.

Two investigators(HL and HH) assessed the risk of bias and clinical applicability of the included studies respectively, using the Quality Assessment of Diagnostic Accuracy Studies-2 (QUADAS-2) [14]. The tool comprises four main components: patient selection, trials to be evaluated, reference standard, case flow, and progression. Disagreements were resolved between the two investigators through negotiation or by involving a third reviewer.

### Data extraction

Two investigators(HL and HH) independently extracted data from the eligible studies, including the name of the first author, year of publication, country, device, study design, analysis, patient characteristics, radiotracers dose, interpretation criteria of PET or PET/CT, cutoff values, sensitivity, specificity, and area under the curve (AUC) with 95% confidence interval (CI).

### Risk of bias

The quality of the included studies was evaluated using QUADAS-2 in Review Manager 5.4. Deeks’ funnel plot was constructed to evaluate the risk of publication bias across all included studies.

### Statistical analysis

The pooled sensitivity, specificity, positive likelihood ratio (PLR), negative likelihood ratio (NLR), diagnostic ratio (DOR), and the corresponding 95% confidence interval (CI) were calculated by extracting the TP, FP, FN, and TN values. Summary receiver operating characteristic (SROC) curves were plotted to calculate the AUC and test the pooled diagnostic value of PET or PET/CT. The heterogeneity between studies was assessed using the chi-square and I^2^ test [15], whereas for quantitative analysis, h it was assessed using Cochran’s Q test and I^2^ test. *P*-value was less < than 0.05 and >50%, for Cochran’s Q test, and I^2^ test, respectively, indicating significant heterogeneity between studies and the selection of random-effect model [16, 17]. A bivariate random-effects model was employed for this synthesis, as it accounts for the potential trade-off and negative correlation between sensitivity and specificity across studies. We performed a subgroup analysis to determine the source of heterogeneity. To determine the reliability of the meta-analysis results a sensitivity analysis was conducted. Deeks’ funnel plots were used to evaluate publication bias, with *p* value of less than 0.10 indicating significant asymmetry and potential bias [18]. The clinical utility of PET or PET/CT was further illustrated using Fagan’s nomogram to calculate post-test probabilities based on the pooled likelihood ratios, assuming a pre-test probability of 50%. Additionally, the diagnostic efficacy of PET and PET/CT was assessed by Fagan’s nomogram. Review Manager 5.4 was used to evaluate the quality of the literature, and Stata 16.0 was used to analyze all data. Statistical significance was set at *P*-value < 0.05. significant.

## Results

### Study selection

**Fig. 1:**
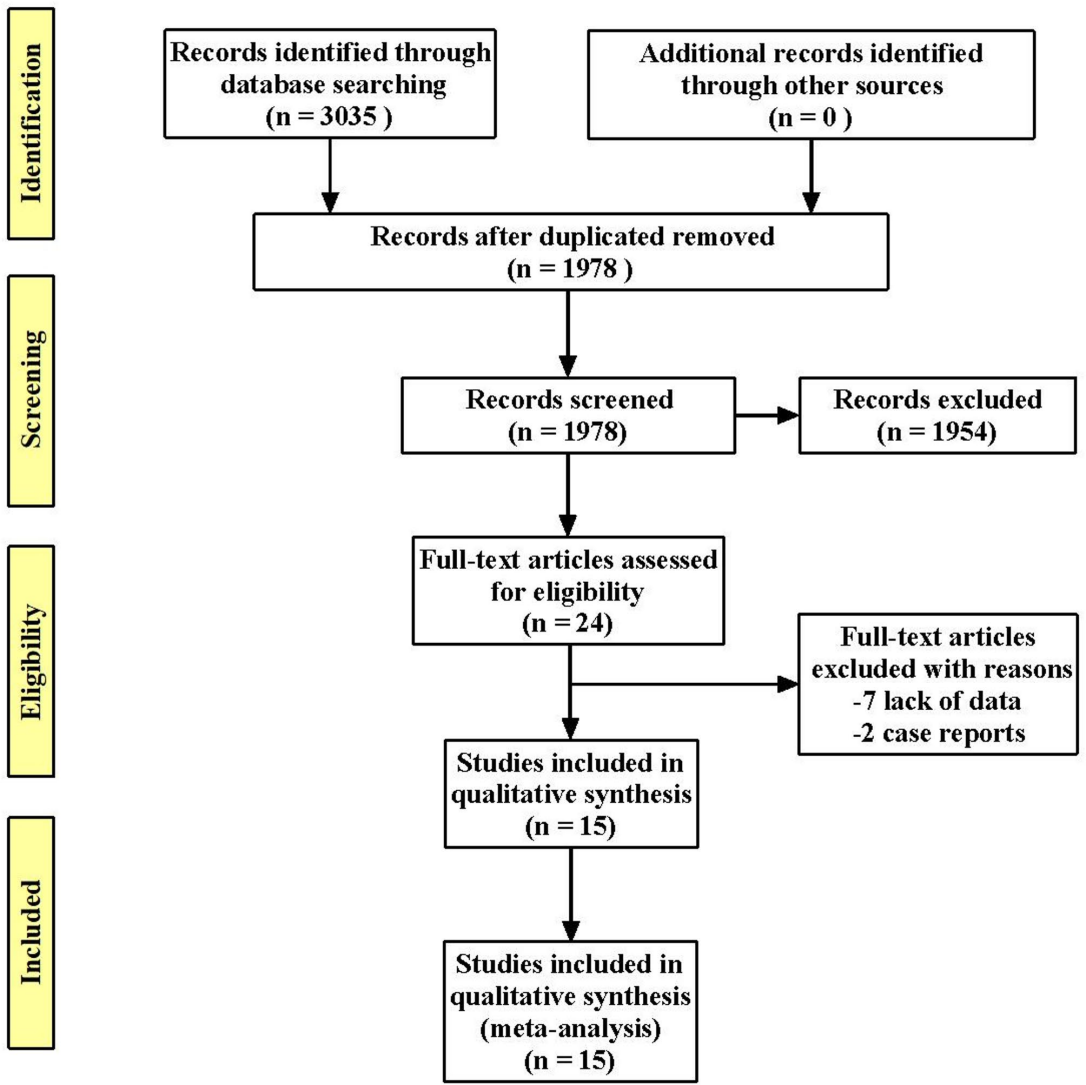
Flow diagram showing selection of studies for meta-analysis

A total of 3035 records were retrieved from the PubMed, Cochrane Library, Embase, and Web of Science databases. Following exclusion, 1057 duplicate studies and 1954 irrelevant studies, reviews, conference abstracts, and case reports were excluded. Twenty-four full-text articles were evaluated for eligibility. Of these, nine studies were excluded, including seven studies with insufficient data and two case report. Consequently, 15 studies were included in the meta-analysis. The screening flowchart is shown in Fig. 1.

Totally, 15 patient-based studies (spanning 2007–2025) were included in this meta-analysis (Table 1). Among these studies, we used PET/CT as the imaging device for ten, and the other studies used PET in combination with other imaging devices. Regarding the interpretation criteria of PET or PET/CT, 12 studies were diagnosed with SUVmax, one study used a ring shape index, another study reported that SUVmax at one hour post-contrast injection had significantly higher value in distinguishing different risk groups, and the remaining study did not provide an internal reference. All the cases included in the analysis were primary disease, and had only received preoperative ^18^F-FDG PET.

**Table 1:** Characteristics of the included studies

First author	Years	Country	Device	Analysis	patient	age	M/F	Primary tumour	Tracer	Injected activity	Interpretation criteria of PET or PET/CT	cut -off	study design	Sen (%)	Spe (%)	AUC
Cho MH [19]	2015	Korea	PET/CT	PB	40	59	18/22	18 in stomach, 22 in intestine	18 F-FDG	5.5MBq/kg	SUVmax	4.99	P	89.5	76.2	0.875
Park JW [20]	2011	Korea	PET/CT	PB	26	62	10/16	26 in stomach	18 F-FDG	200MBq	SUVmax	3.94	R	85.7	94.7	0.872
Yamada M [21]	2007	Japan	PET	PB	21	62	13/8	21 in stomach	18 F-FDG	200MBq	SUVmax	6.5	R	100	100	NA
Kaneta T [22]	2009	Japan	PET	PB	41	60.3	24/17	41 in stomach or small intestine	18 F-FDG	185-370MBq	NA	NA	R	NA	NA	NA
Tokumoto N [23]	2014	Japan	PET/CT	PB	30	66	16/14	30 in stomach	18 F-FDG	200MBq	SUVmax	3	R	85.7	62.5	NA
Yoshikawa K [24]	2014	Japan	PET/CT	PB	10	62.5	7/3	6 in stomach, 3 in intestine, 1 in rectum	18 F-FDG	3.7MBq/kg	SUVmax	5	R	NA	NA	NA
Li S [25]	2022	China	PET/CT	PB	26	60.85 ± 9.37	13/13	26 in stomach	18 F-FDG	185-370MBq	SUVmax	2.6	R	100	83.3	0.967
Narushima K [26]	2023	Japan	PET	PB	89	65	41/48	2 in esophagus, 68 in stomach, 9 in small bowel, 10 in colorectum	18 F-FDG	370MBq	SUVmax	5.68	R	87	86.4	0.905
Albano D [27]	2020	Italy	PET/CT	PB	54	64.7	30/24	29 in stomach, 19 in small intestine, 2 in rectum, 2 in cecum, 2 in omentum	18 F-FDG	3.5–4.5.5MBq/kg	SUVmax	NA	R	89	97	NA
Yoo J [28]	2020	Korea	PET/MRI	PB	18	NA	NA	18 in stomach	18 F-FDG	5.2 MBq/kg	SUVmax	5.6	R	80	66.7	0.667
Kwon Y [29]	2019	Korea	PET/CT	PB	32	60.1	14/18	31 in stomach, 1 in small intestine	18 F-FDG	370–480 MBq	SUVmax at 1 h postcontrast injection	5.2	R	80	89	0.863
Miyake KK [30]	2016	Japan	PET/CT	PB	46	≥ 60	27/19	2 in oesophagus, 30 in stomach, 12 in small intestine, 2 in colon/rectum	18 F-FDG	294 ± 82MBq	Ring-shaped/Not ring-shaped	NA	R	NA	NA	NA
Kurata Y [31]	2018	Japan	CECT/PET	PB	64	65	36/28	49 in stomach, 6 in duodenum, 4 in small intestine, 3 in colon and rectum, 2 in esophagus	18 F-FDG	250MBq/60kg	SUVmax	5.88	R	85.7	90.3	0.93
Kawabata K [32]	2025	Japan	PET/CT	PB	45	61	27/18	20 in stomach, 23 in small intestine, 2 in rectum	18 F-FDG	3.7 MBq/kg	SUVmax	5	R	1	1	NA
Wang R [6]	2025	China	PET/CT	PB	26	56	7/19	16 in stomach, 8 in small intestine, 2 in peritoneal cavity	18 F-FDG	87 ± 31.5 MBq	SUVmax	6	P	0.72	0.857	0.818

*PB* Patient based analysis, *R* Retrospective, *P* Prospective, *Sen* sensitivity, *Spe* specificity, *AUC* area under the curve, *CI* confidence interval

### Quality assessment

**Fig. 2:**
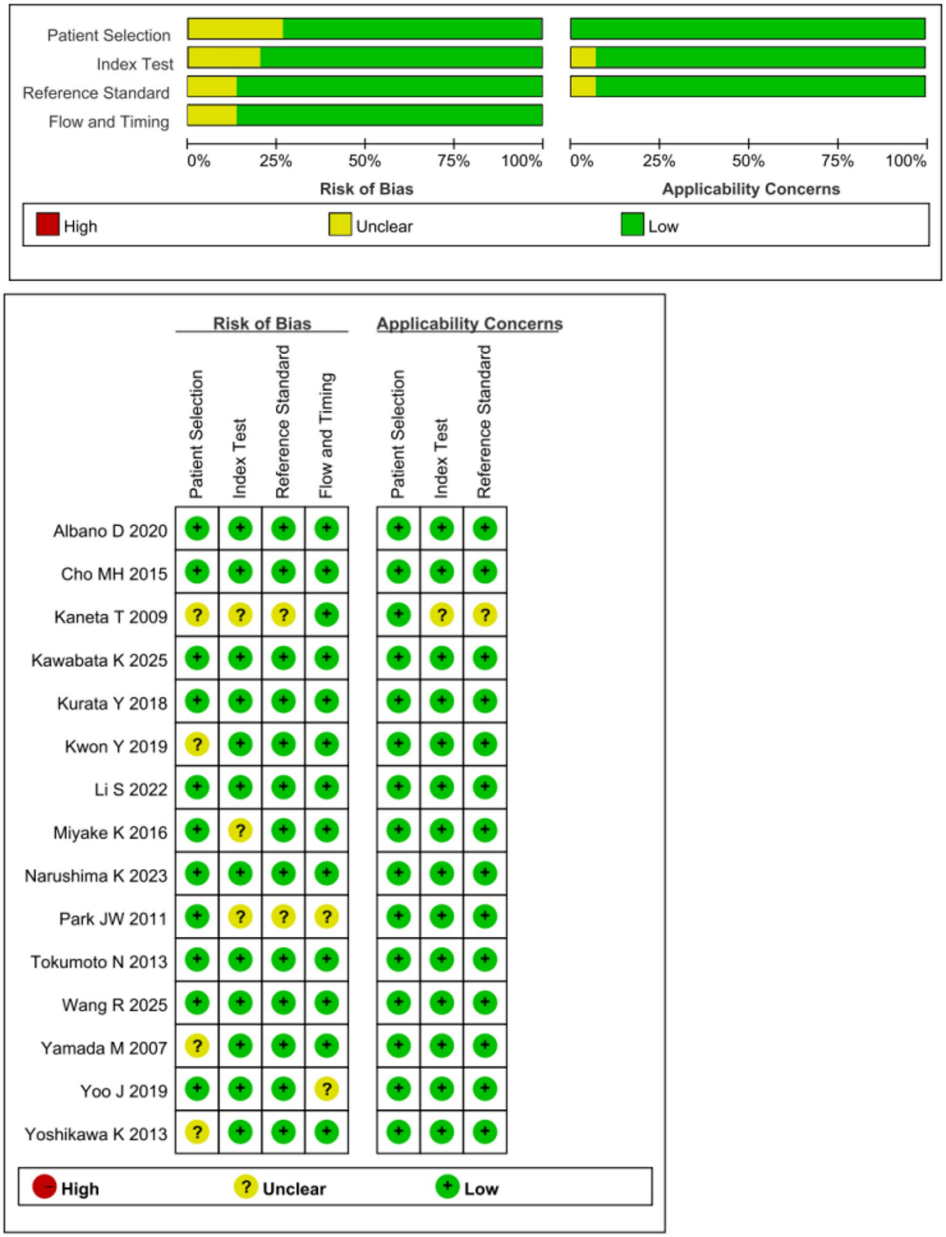
Quality Assessment of Diagnostic Accuracy Studies-2 assessment for risk of bias and applicability. Red, yellow and green indicate high, unclear and low risk respectively

We evaluated the quality of the included studies using QUADAS-2, and the results were analyzed using Review Manager 5.4 as depicted in Fig. 2. Most studies included in our meta-analysis were continuous and time-scaled. Patients with malignant GISTs included in the study were diagnosed by clinically recognized criteria. In most trials, cutoff values were set for favorable sensitivity, specificity, and accuracy in differentiating risk groups in GISTs, which were not pre-specified, introducing high or unclear risks in the test field.

### Diagnostic accuracy of PET in the malignant potentials of gists

**Fig. 3:**
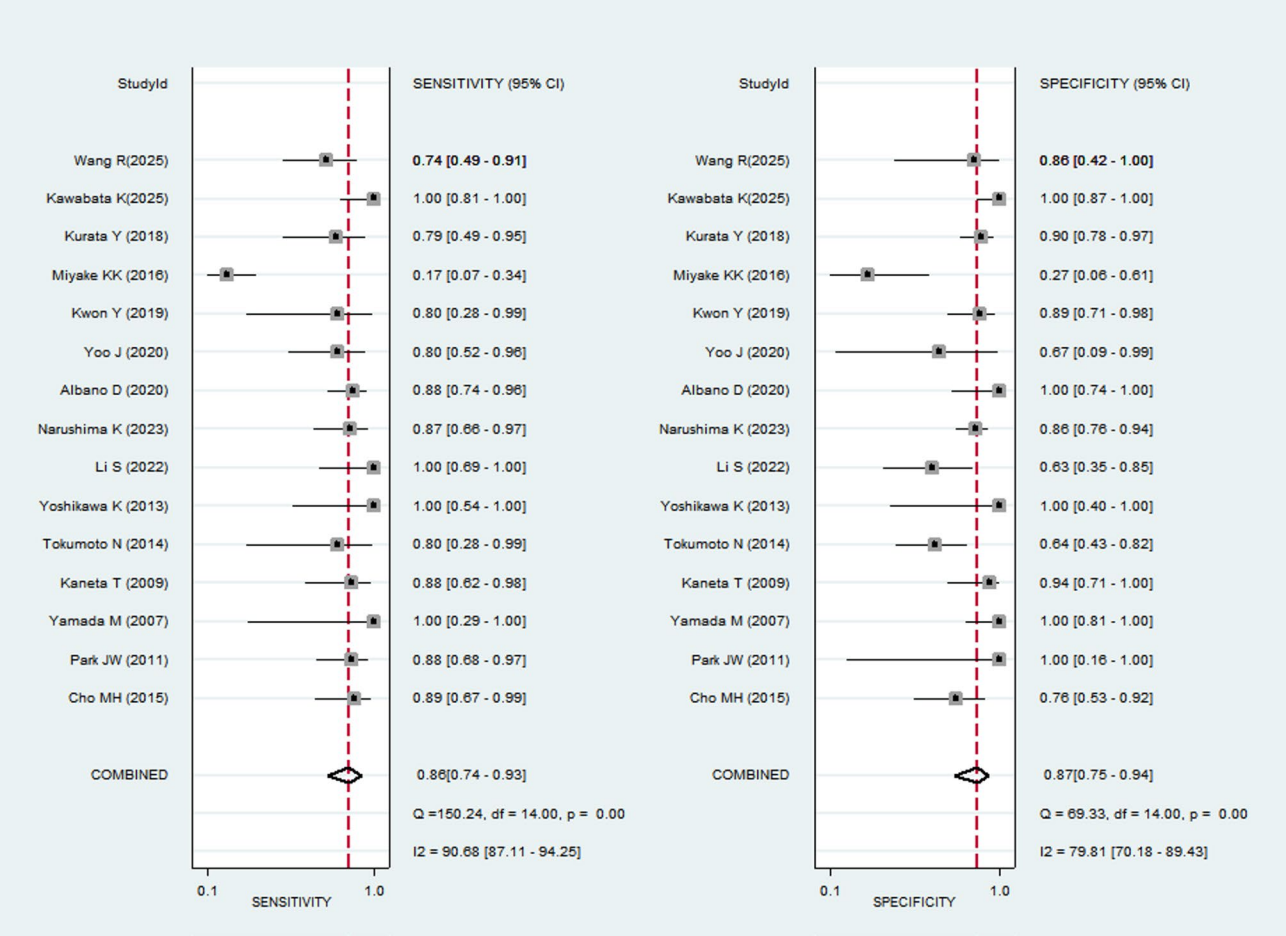
Forest plots of studies examining positron emission tomography used in the diagnosis of malignant potential of Gastrointestinal Stromal Tumors

**Fig. 4:**
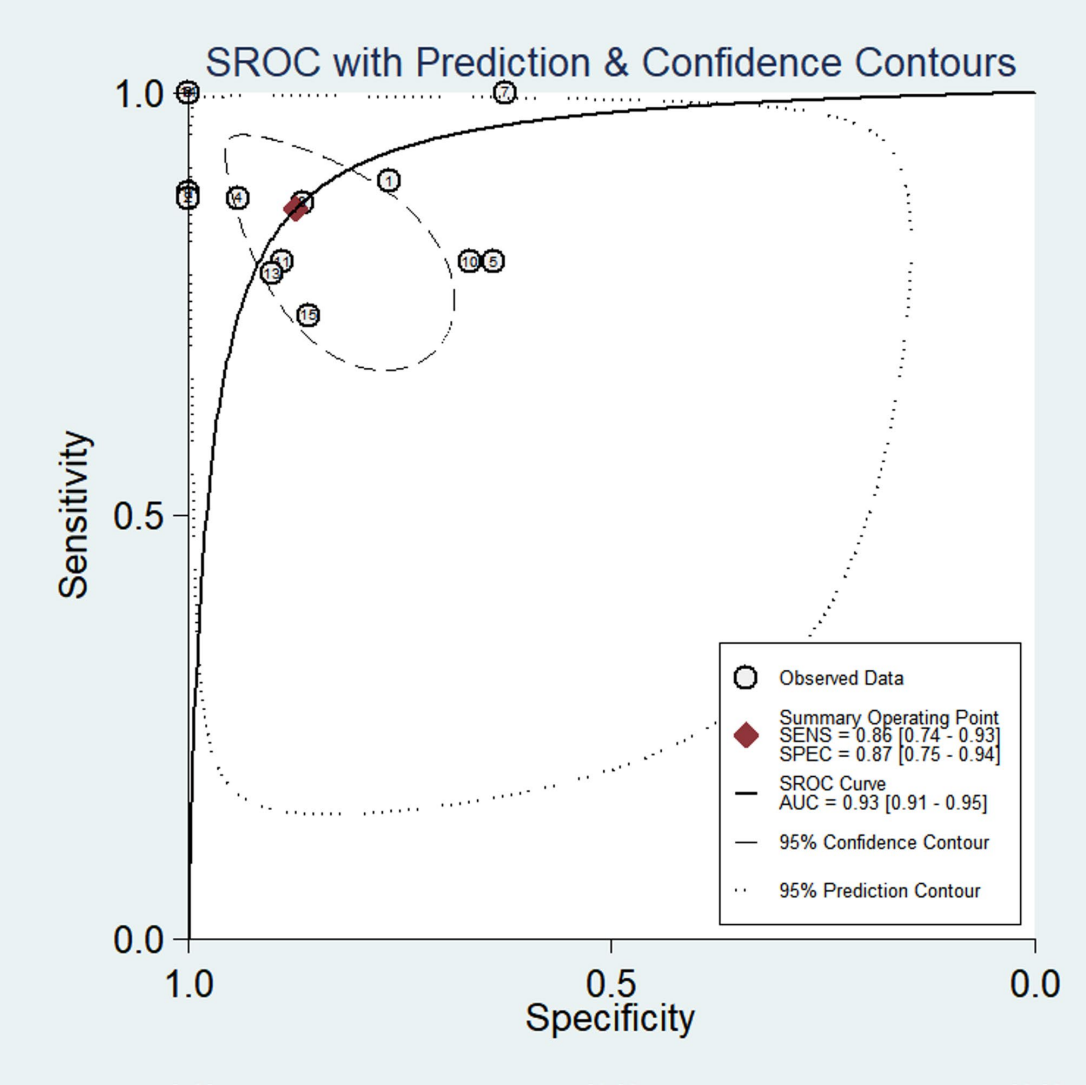
Summary receiver operator characteristic curve examining the overall accuracy of positron emission tomography used in the diagnosis of malignant potential of Gastrointestinal Stromal Tumors

**Fig. 5:**
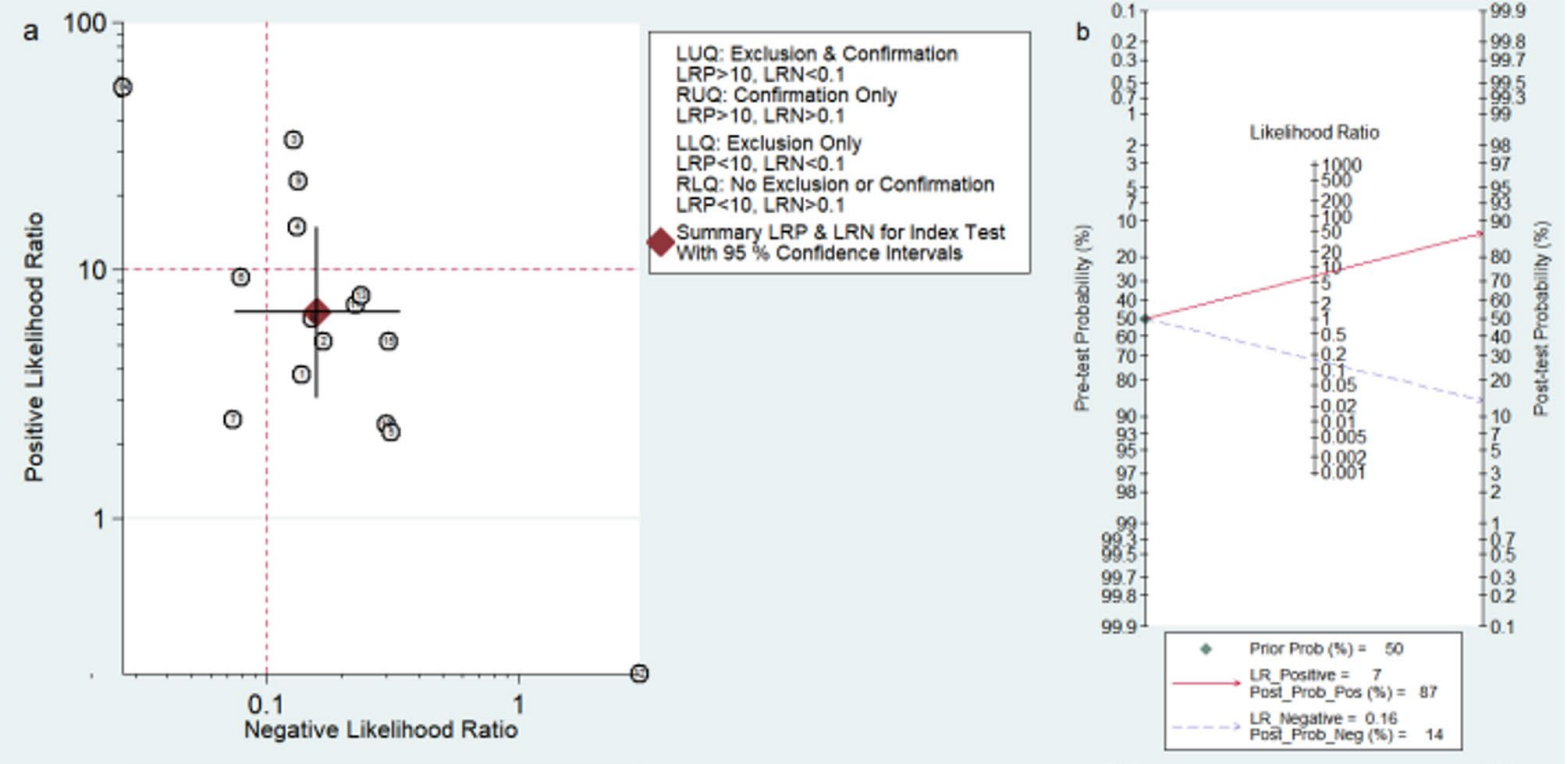
Assessment of clinical applicability of positron emission tomography for diagnosis of malignant potential of Gastrointestinal Stromal Tumors. (**a**) Summary of positive likelihood ratio and negative likelihood ratio for diagnosis of malignant potential of Gastrointestinal Stromal Tumors; (**b**) Fagan’s nomogram of positron emission tomography studies for diagnosis of malignant potential of Gastrointestinal Stromal Tumors

Figure 3 displays the sensitivity and specificity of PET or PET/CT for diagnosing the malignant potential of GISTs. Forest plots of pooled data revealed a large heterogeneity among studies, with I^2^ = 90.68% for sensitivity and I^2^ = 79.81% for specificity. A random-effects model was used to estimate the diagnostic performance of PET or PET/CT in the malignancy of GISTs, yielding the following results: sensitivity, 0.86 (95% CI: 0.74–0.93); specificity, 0.87 (95% CI: 0.75–0.94); PLR, 6.80 (95% CI: 3.09–14.96); NLR, 0.16 (95% CI: 0.07–0.34); DOR, 43.09 (95% CI: 10.02–185.28.02.28). These indicate that PET or PET/CT has significant diagnostic value for distinguishing different risk grades. In addition, to assess the diagnostic accuracy, we plotted the SROC curve (Fig. 4). The AUC was 0.93 (95% CI: 0.91–0.95), indicating a strong overall accuracy of PET in the diagnosis of GISTs. Aiding the clinical decision-making during diagnosis is an important value of imaging devices. Therefore, to confirm its diagnostic accuracy we plotted Fagan’s nomogram with post-test probabilities of 87% and 14% for the PLR and NLR, respectively, with a pre-test probability set at 50% (Fig. 5).

### Subgroup analyses and sensitivity analyses

To investigate potential heterogeneity, we performed a subgroup analysis (Table 2) divided according to publication year, sample size, imaging device, and cutoff value setting.

**Table 2:** Summary estimates of diagnostic power and their 95% confidence intervals. Sen, sensitivity; Spe, specificity; PLR, positive likelihood ratio; NLR, negative likelihood

Subgroup	No. studies	Sen (95% CI)	Spe (95% CI)	PLR (95% CI)	NLR (95% CI)	DOR (95% CI)	AUC (95% CI)	Difference between subgroup
Publicationyear								*p* > 0.05
< 2015	5	0.89 (0.76–0.95)	0.95 (0.61–1.00.61.00)	19.37 (1.60–234.27.60.27)	0.11 (0.05–0.27)	168.98 (9.37–3047.92.37.92)	0.93 (0.90–0.95)	
≥ 2015	10	0.83 (0.66–0.93)	0.84 (0.69–0.93)	5.23 (2.25–12.17)	0.20 (0.08–0.48)	26.49 (5.00–140.35.00.35)	0.91 (0.88–0.93)	
samplesize								*p* > 0.05
< 40	8	0.85 (0.76–0.91)	0.85 (0.67–0.94)	5.64 (2.37–13.40)	0.18 (0.10–0.30)	32.03 (9.99–102.67.99.67)	0.86 (0.82–0.89)	
≥ 40	7	0.85 (0.60–0.96)	0.88 (0.68–0.96)	7.30 (2.01–26.55)	0.17(0.05–0.60)	42.61 (3.55–511.58.55.58)	0.93(0.91–0.95)	
cutoff								*p* < 0.05
< 5	4	0.90 (0.79–0.95)	0.69 (0.56–0.79)	2.87 (1.97–4.17)	0.15 (0.07–0.33)	19.07 (7.04–51.66)	0.88 (0.85–0.91)	
≥ 5	8	0.89 (0.74–0.95)	0.92 (0.83–0.97)	11.43 (4.66–28.03)	0.12 (0.05–0.31)	91.72 (18.17–462.93.17.93)	0.96 (0.94–0.97)	
device								*p* < 0.05
PET	5	0.85 (0.74–0.91)	0.90 (0.84–0.94)	8.13 (5.06–13.07)	0.17 (0.10–0.30)	47.05 (20.61–107.39.61.39)	0.94 (0.91–0.95)	
PET/CT	10	0.87 (0.67–0.95)	0.85 (0.63–0.95)	5.61 (1.91–16.50)	0.16 (0.05–0.49)	35.66 (4.47–284.81.47.81)	0.92 (0.90–0.94)	

ratio, *DOR* diagnostic odds ratio, *AUC* area under the curve, *CI* confidence interval

**Fig. 6:**
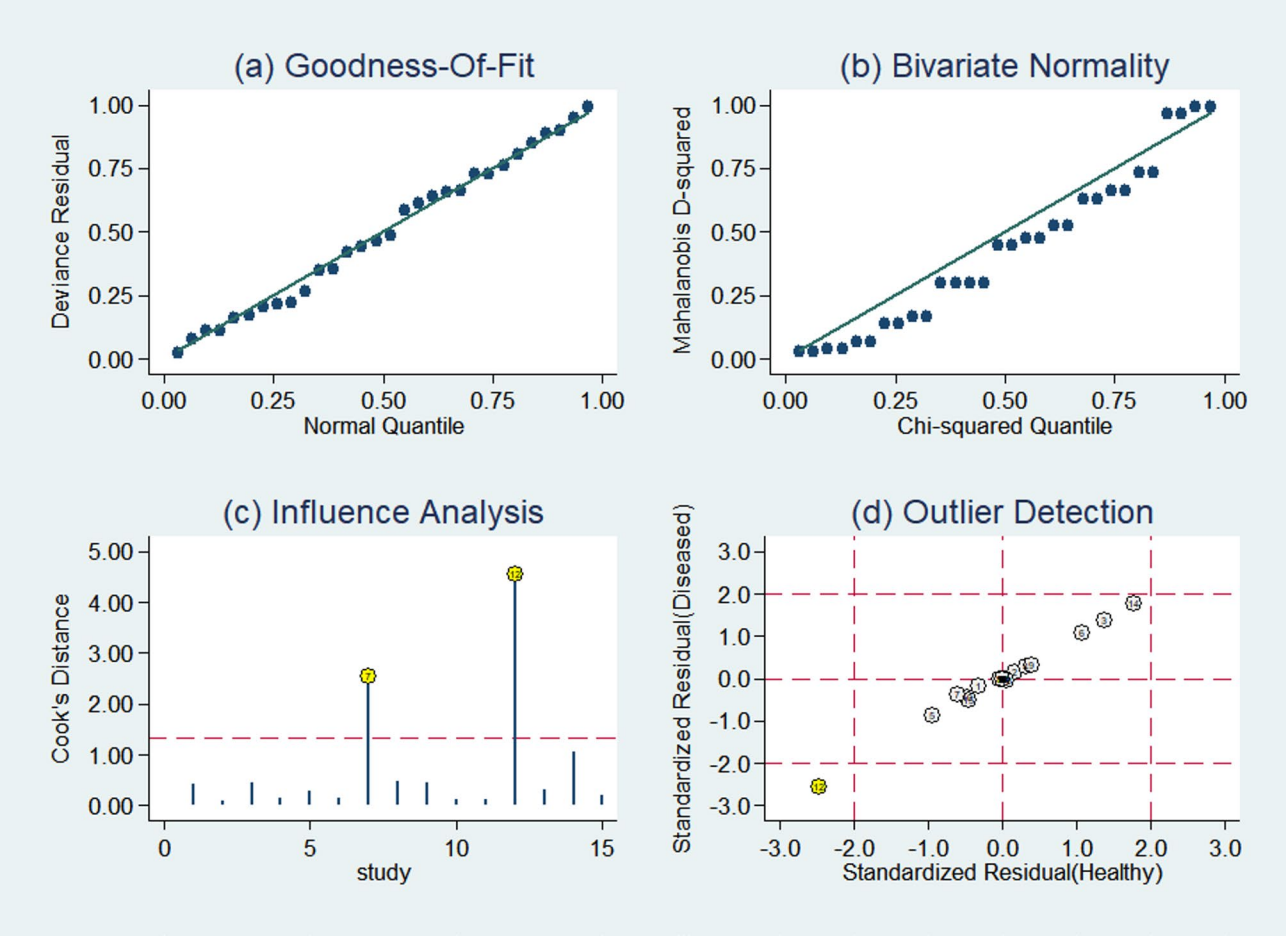
Diagram of sensitivity analysis. (**a**) goodness-of-fit; (**b**) bivariate normality; (**c**) influence analysis; (**d**) outlier detection

The results of the sensitivity analysis are shown in Fig. 6. The goodness of fit (Fig. 6A) and bivariate normality (Fig. 6B) indicated the suitability of the random-effects model. The influence analysis revealed that the studies by Li et al. and Miyake et al. were the most dominant in terms of weight (Fig. 6C). Outlier detection implied that heterogeneity might be attributed to data from Miyake et al. (Fig. 6D). Following the exclusion of one outlier study by Miyake et al. which used ring-shaped/not ring-shaped as an internal reference rather than SUVmax (Table 3), the sensitivity and specificity of the heterogeneous I^2^ changed significantly.

**Table 3:** Diagnostic performance of positron emission tomography in the diagnosis of malignant potiential of Gastrointestinal stromal tumors

Analysis	Overall	Outliers excluded
No.of studies	15	14
Sen (95% CI)	0.86 (0.74–0.93)	0.87 (0.82–0.91)
Spe (95% CI)	0.87 (0.75–0.94)	0.89 (0.80–0.95)
PLR (95% CI)	6.80 (3.09–14.96)	8.24 (4.11–16.55)
NLR (95% CI)	0.16 (0.07–0.34)	0.14 (0.10–0.21)
DOR (95% CI)	43.09 (10.02–185.28.02.28)	57.28 (22.64–144.90)
AUC (95% CI)	0.93 (0.91–0.95)	0.91 (0.88–0.93)

*Sen* sensitivity, *Spe* specificity, *PLR* positive likelihood ratio, *NLR* negative likelihood ratio, *DOR* diagnostic odds ratio, *AUC* area under the curve, *CI* confidence interval

Notably, the diagnostic accuracy was higher when the cut-off values were set at ≥ 5, compared with < 5. The sensitivity, specificity, PLR, NLR, DOR, and AUC values for cut-off values ≥ 5 and < 5 were 0.89 (95% CI: 0.74–0.95), 0.92 (95% CI: 0.83–0.97), 11.43 (95% CI: 4.66–28.03), 0.12 (95% CI: 0.05–0.31), 91.72 (95% CI: 18.17–462.93.17.93), 0.96 (95% CI: 0.91–0.95), 0.90 (95% CI: 0.79–0.95), 0.69 (95% CI: 0.56–0.79), 2.87 (95% CI: 1.97–4.17), 0.15 (95% CI: 0.07–0.33), 19.07 (95% CI: 7.04–51.66), and 0.88 (95% CI: 0.85–0.91), respectively. Therefore, higher cutoff values may have better diagnostic accuracy in distinguishing high-risk from other risk groups.

The results of the diagnostic accuracy of malignant potentials of GISTs for sample size < 40 and ≥ 40 are listed as follows: the sensitivity was 0.85 (95% CI: 0.76–0.91) vs. 0.85 (95% CI: 0.60–0.96), specificity was 0.85 (95% CI: 0.67–0.94) vs. 0.88 (95% CI: 0.68–0.96), PLR was 5.64 (95% CI: 2.37–13.40) vs. 7.30 (95% CI: 2.01–26.55), NLR was 0.18 (95% CI: 0.10–0.30) vs. 0.17 (95% CI: 0.05–0.60), DOR was 32.03 (95% CI: 9.99–102.67.99.67) vs. 42.61 (95% CI: 3.55–511.58.55.58), and AUC was 0.86 (95% CI: 0.82–0.89) vs. 0.93 (95% CI: 0.91–0.95). A significant difference was not observed in the overall diagnostic accuracy for malignancy in GISTs for sample size < 40 compared with sample size ≥ 40. In addition, publication year has not shown good impact on the diagnostic value of PET/CT, for *p* value > 0.05, the differences between studies published before and after 2015 were not significant, actually. In the group diagnosing with PET/CT, they had pooled results for sensitivity, specificity, PLR, NLR, DOR and AUC of 0.87 (95%CI: 0.67–0.95), 0.85 (95%CI: 0.63–0.95), 5.61 (95%CI: 1.91–16.50), 0.16 (95%CI: 0.05–0.49), 35.66 (95%CI: 4.47–284.81.47.81) and 0.92 (95%CI: 0.90–0.94). Conversely, studies using PET combined with other devices showed slightly higher diagnostic value with sensitivity of 0.85 (95%CI: 0.74–0.91), specificity of 0.90 (95%CI: 0.84–0.94), PLR of 8.13 (95%CI: 5.06–13.07), NLR of 0.17 (95%CI: 0.10–0.30), DOR of 47.05 (95%CI: 20.61–107.39.61.39), and AUC of 0.94 (95%CI: 0.91–0.95), respectively. Overall, although there were minor differences in the diagnostic accuracy between CT and other devices, preoperative PET demonstrated excellent accuracy in differentiating the high-risk from the other risk groups before surgery.

### Publication bias

**Fig. 7:**
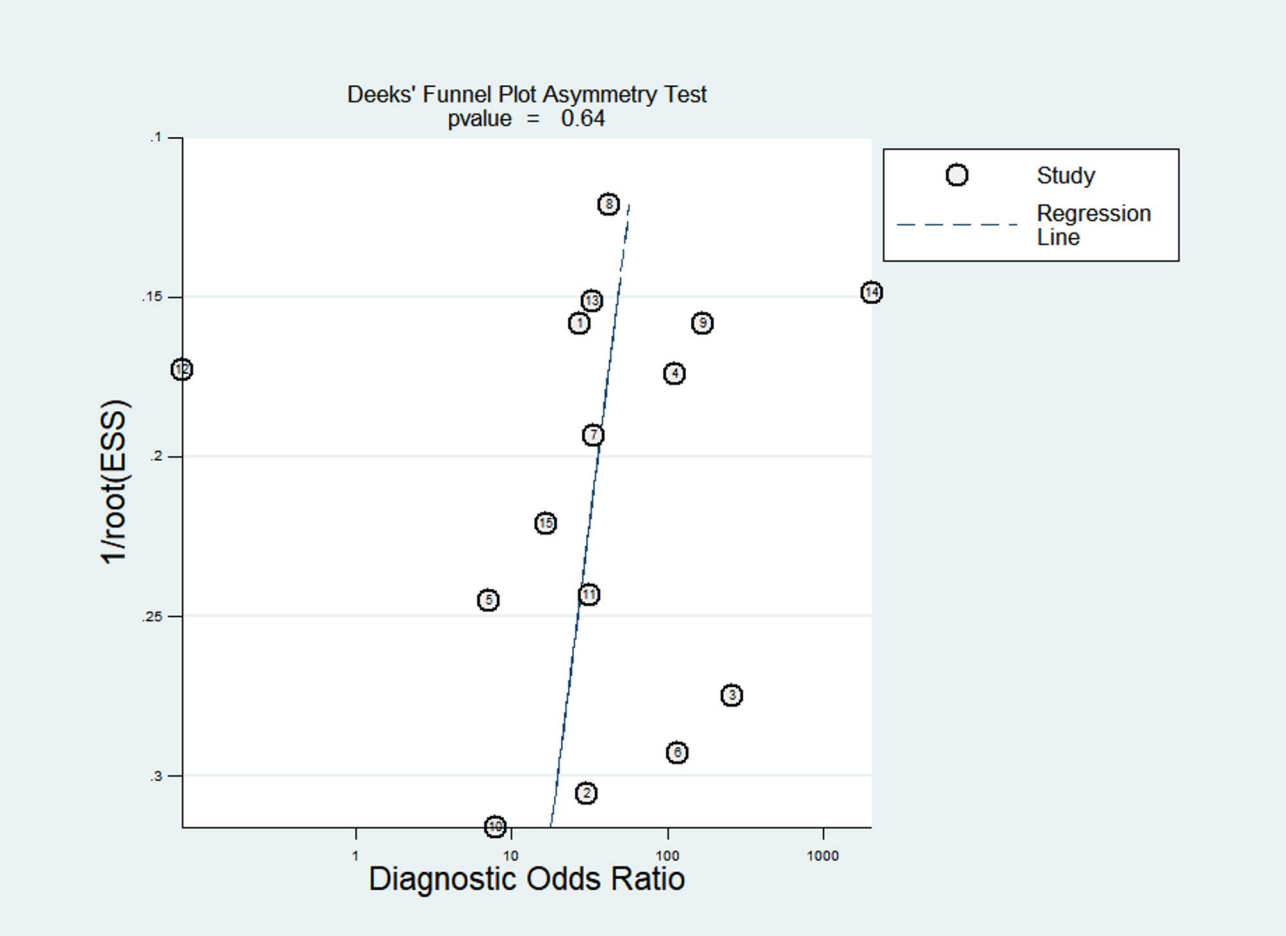
Funnel plot for determining publication bias

Deeks’ funnel plot was generated for the 15 studies included (Fig. 7). The studies were symmetrically distributed on both sides of the regression line (*p* = 0.64), and the differences were not statistically significant, indicating no publication bias in the included data.

## Discussion

PET, as a non-invasive imaging modality, has emerged as a transformative tool in oncology [33, 34]. For GISTs, several studies have used PET or PET/CT to detect the efficacy of treatment after using imatinib or other tyrosine kinase inhibitors (TKIs) [35–37], The metabolic responses of ^18^F-FDG observed on PET are closely related to clinical benefits [38]. This was properly conducted and evaluated by a meta-analysis [39]. Additionally, several studies have also shown that postoperative PET has clinical value in the restaging of GIST, to evaluate the possibility of recurrence and metastasis [40, 41]. However, its preoperative role in malignant diagnosis and prognosis of GISTs remains underexplored.

Currently, the standard treatment for patients with localized GISTs is complete surgical resection, and ^18^F-FDG PET/CT whole-body imaging can effectively display the primary lesion and possible metastatic lesion, and help to judge the scope of the lesion, which is conducive to guiding the mode of surgery and choosing the time of surgery, and has an important influence on treatment decision [42]. In terms of surgical implementation, PET/CT can accurately reflect the lesion scope and determine the surgical field for patients with good adjuvant treatment effect [43]. For those with poor results, PET can detect disease progression early so that surgeons can take palliative surgery in time. Besides, postoperative patients at high risk of recurrence usually require adjuvant TKI therapy. However, risk classification factors in the current guidelines such as mitotic count, tumor size, and surgical resection margin, are usually only available after surgery, preoperative risk stratification of GISTs remains a clinical challenge [26]. While biopsy and mitotic counting are gold standards, many cases are infeasible or yield insufficient tissue endoscopic biopsy, non-invasive imaging methods and volumetric parameters of PET may help predict prognosis before treatment and guide pathologists in locating areas that might have high Ki-67 values, resulting in a more accurate assessment of patient prognosis.

In this study, we included 15 articles with a total of 568 patients with localized primary GISTs. Our results demonstrated that preoperative PET demonstrated high sensitivity (0.86, 95% CI: 0.74–0.93) and specificity (0.87, 95% CI: 0.75–0.94) in the diagnosis of different risk groups in GISTs, with an AUC of 0.93. These findings are consistent with previous research. For instance, Yamada et al. [21] also reported high diagnostic accuracy (100% sensitivity and specificity) using PET with an SUVmax cutoff of 6.5, suggesting that GISTs with malignant potential exhibit higher metabolic activity leading to greater radiotracer accumulation, such as FDG [38, 44].

Among all these studies, three analyzed the prognostic value of preoperative PET in patients with localized primary GISTs. Miyake et al. [30] found that ring-shaped uptake on preoperative ^18^F-FDG PET/CT was a significant prognostic factor for localized primary GISTs. However, the above parameters could not be quantitatively analyzed, affecting the sensitivity and specificity of heterogeneous I^2^ significantly, with the results being consistent with our sensitivity analysis. Total lesion glycolysis (TLG) can measure tumor load, which reflects both tumor metabolic activity and tumor metabolic volume. And metabolic tumor volume (MTV), another measurement derived from ^18^F-FDG PET/CT, is defined as the volume of tumor showing increased FDG uptake and is a more recently investigated FDG PET/CT index measurement. MTV represents the extent of FDG uptake by a tumor, which has been suggested as an independent prognostic indicator of clinical outcome for certain cancers [44]. Albano et al. [27] identified preoperative MTV and TLG as independent prognostic factors for localized primary GISTs. These findings were also supported by the studies by Hwang et al. [38], which also showed that MTV and TLG have a high predictive prognostic value for RFS in patients with localized primary GISTs. Similarly, Narushima et al. [26] used SUVmax to evaluate the malignant potential and prognostic value of localized primary GISTs, and found that tumor size, mitotic count and Ki-67 showed significant positive correlations with SUVmax, when SUVmax was set as 5.68, there is good diagnostic accuracy for the high-risk group, the death group and the relapse group. Our results demonstrated that preoperative PET demonstrated high sensitivity (0.86, 95% CI: 0.74–0.93) and specificity (0.87, 95% CI: 0.75–0.94) in the diagnosis of different risk groups in GISTs. Our results show that PET has a significant diagnostic value for assessing the malignant potential of GISTs.

Owing to the heterogeneity among the included studies, we conducted a subgroup analysis to explore confounding factors, and there were several notable findings. For example, when the cut-off values were ≥ 5, the diagnostic accuracy was higher than when the cut-off was < 5. This might be attributed to the accumulation of radiotracers in GISTs with malignant potentials, this finding was correlated with Du et al. [45], who indicated that glucose metabolism is abnormally increased in GISTs with vigorous mitosis and higher biological risk leading to higher FDG accumulation. Furthermore, though there was a little difference between the PET and PET/CT groups, the diagnostic value of sensitivity in the PET/CT group was better than that of PET, which may be attributed to missed diagnosis of PET was mainly due to the insignificant uptake of imaging agents in some lesions or it was easy to be mistaken for physiological uptake. The anatomical features of CT can effectively make up for this shortcoming. Overall, PET combined with other devices group had better accuracy, this was because CECT, MRI or other imaging equipment had higher resolution radios than CT, thus, it had a greater advantage in the aspect of being identified by imaging experts. However, due to the majority of studies have adopted the diagnostic model of PET combined with CT, and there were relatively few studies that combine other imaging devices, the results may be related to the heterogeneity between studies and small sample size. Consequently, preoperative PET findings are of great value in the prognostic analysis of patients with GISTs. Additionally, PET-related parameters may be better than the revised NIH criteria in predicting RFS for inhomogeneous mitotic counts in larger GISTs, where metabolic indices can be easily obtained [46]. Overall, preoperative PET showed excellent clinical significance and guiding value in prognostic analysis.

The choice of radiotracer might also influence diagnostic performance. While our analysis predominantly included ^18^F-FDG studies (14/15), one study also evaluated ^68^Ga-RM26 [6]. Emerging tracers like ^68^Ga-FAPI-04 have shown promise, potentially offering higher tumor-to-background ratios in some cases [7]. However, the current evidence base for non-FDG tracers in preoperative diagnosis remains limited, precluding meaningful subgroup analysis. Future studies are needed to compare their diagnostic efficacy against ^18^F-FDG.

This meta-analysis has several strengths and clinical enlightenments. First of all, the preoperative decision-making process in GISTs might be changed. The assessment of malignant potential of GIST relies on postoperative pathology, and preoperative biopsy has some limitations such as risk of bleeding and insufficient tissue volume. This study confirms that preoperative PET or PET/CT can noninvasively predict malignant potential using metabolic parameters such as SUVmax, providing a key decision basis for patients who cannot be biopsy-proven. Meanwhile, preoperative PET or PET/CT may help to optimize surgical planning, PET whole-body imaging can detect both primary and metastatic lesions, which can help to determine the extent of surgery and whether neoadjuvant therapy is necessary to avoid secondary surgery. Besides, our meta-analysis indicates pooled sensitivity 86% (95%CI: 0.74–0.93), specificity 87% (95%CI: 0.75–0.94), AUC = 0.93, which confirmed that PET or PET/CT could accurately distinguish high-risk GISTs. When cutoff values ≥ 5, it had the best diagnostic performance to guide clinical practice. Moreover, metabolic parameters may be superior to the traditional NIH classification in optimizing prognostic stratification, combined with machine learning, personalized prognostic prediction tools can be constructed to lay the foundation for the development of radiomics model in the future. Last but not least, our study was methodologically rigorous, we systematically searched four major databases and included 15 studies that followed the PRISMA guidelines and registered PROSPEO (CRD42024545100), which significantly reduced selection bias. For statistical purposes, a random effects model was used to deal with high heterogeneity, and subgroup analysis was used to explore the sources of heterogeneity in depth to ensure the comprehensiveness and rigor of the analysis.

The limitations of this meta-analysis should be listed. Firstly, the cutoff values of SUVmax in the included studies differed, which may have contributed to heterogeneity. Currently, most studies obtained the SUVmax value with the highest diagnostic efficiency by analyzing the clinical data of GISTs who underwent PET or PET/CT examination. In the research conducted by Kawabata et al. [32], they preset the SUVmax value to classify GISTs as benign and malignant according to the existing studies. The SUVmax value with the highest diagnostic efficiency may be related to the tumor anatomical subtype (e.g., gastric vs. rectal GISTs), so multi-center studies are expected to verify. Besides, SUVmax is not the only factor associated with the revised NIH consensus criteria for prognostic analysis; other volumetric factors, such as MTV or TLG, are also associated and may better predict patient outcomes than NIH classifications, machine learning may assist in constructing the optimal prediction model by analyzing a variety of volume parameters, and strengthen the improvement of diagnostic performance. Furthermore, apart from ^18^F FDG, there have been few studies on the use of other radiotracers to predict the malignant potential of GISTs, which may due to clinical application and other considerations. Though other radiotracers have shown better diagnostic efficacy, the sample size and the number of studies may still have limited the statistical power. Moreover, different risk classifications can also be a source of heterogeneity. The study conduncted by Yamada et al. [21] used the NIH standard as the risk classification criterion, while the other studies included in the analysis used the modified NIH standard. Therefore, there may also be some heterogeneity. Lastly, most studies were retrospective case controls, increasing the risk of bias in the quality assessment of the patient selection domains. Therefore, based on the above limitations, these findings need to be interpreted prudently, and the results of our meta-analysis need to be further confirmed by well-designed studies with larger sample sizes in the future.

## Conclusion

In summary, our meta-analysis demonstrated the excellent diagnostic performance of preoperative PET in assessing the malignant potential of GISTs as an effective non-invasive imaging device. Preoperative PET findings may aid in the preoperative preparation and prognostic classification in patients with localized primary GISTs. Furthermore, prospective, well-designed studies with large sample sizes are required to validate our results and confirm the clinical value of preoperative PET in the diagnosis of the malignant potential of GISTs.

## Supplementary Information

Supplementary Material 1.

## Data Availability

Data sharing is not applicable to this article as no datasets were generated or analysed during the current study.

## Abbreviations

AUC: Area Under the Curve
CI: Confidence Interval
CT: Computed Tomography
DOR: Diagnostic Odds Ratio
EUS: Endoscopic Ultrasonography
ESMO: European Society for Medical Oncology
FDG: Fluorodeoxyglucose
FAPI: Fibroblast Activation Protein Inhibitor
FN: False Negative
FP: False Positive
GIST(s): Gastrointestinal Stromal Tumor(s)
ICC: Interstitial Cells of Cajal
MRI: Magnetic Resonance Imaging
MTV: Metabolic Tumor Volume
NA: Not Available
NCCN: National Comprehensive Cancer Network
NLR: Negative Likelihood Ratio
NIH: National Institutes of Health
PB: Patient-Based (Analysis)
PET: Positron Emission Tomography
PET/CT: Positron Emission Tomography-Computed Tomography
PLR: Positive Likelihood Ratio
PRISMA: Preferred Reporting Items for Systematic Reviews and Meta-Analyses
PROSPERO: International Prospective Register of Systematic Reviews
QUADAS-2: Quality Assessment of Diagnostic Accuracy Studies-2
RFS: Recurrence-Free Survival
ROC: Receiver Operating Characteristic
SEN: Sensitivity
SPE: Specificity
SROC: Summary Receiver Operating Characteristic
SUVmax: Maximum Standardized Uptake Value
TLG: Total Lesion Glycolysis
TKI(s): Tyrosine Kinase Inhibitor(s)
TN: True Negative
TP: True Positive
