# Supplementary figures and images for: Preoperative PET or PET/CT for malignant diagnosis of Gastrointestinal stromal tumors: a systematic review and meta-analysis
Source: BMC Cancer. 2026 Mar 31;26:590. doi: 10.1186/s12885-025-15278-3 (PMC13159186; doi:10.1186/s12885-025-15278-3)

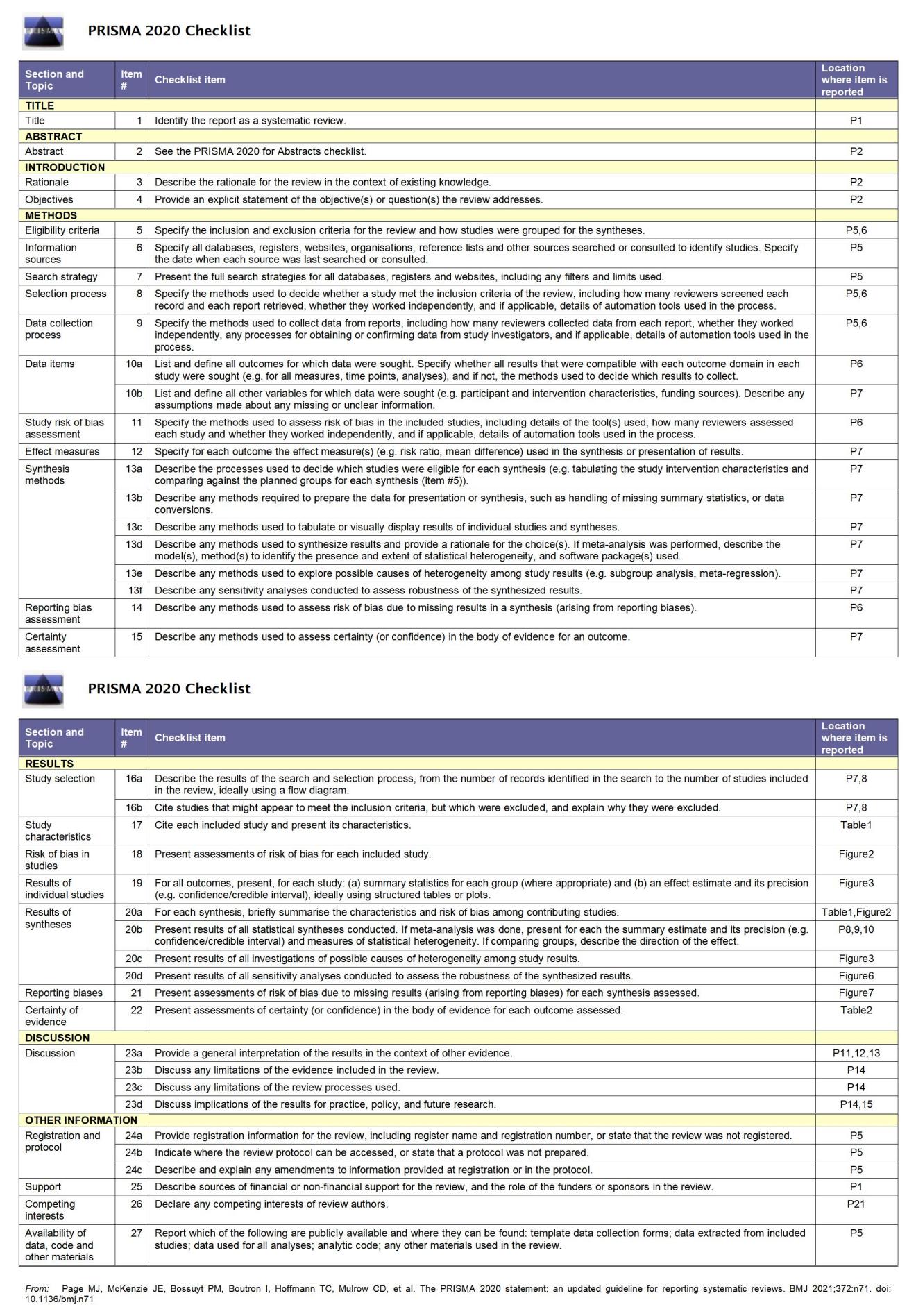

Supplement: Supplementary file 1 — Supplementary Material 1. [file 12885_2025_15278_MOESM1_ESM.docx]
